# Reducing Symptom Distress in Patients With Advanced Cancer Using an e-Alert System for Caregivers: Pooled Analysis of Two Randomized Clinical Trials

**DOI:** 10.2196/jmir.7466

**Published:** 2017-11-14

**Authors:** David H Gustafson, Lori L DuBenske, Amy K Atwood, Ming-Yuan Chih, Roberta A Johnson, Fiona McTavish, Andrew Quanbeck, Roger L Brown, James F Cleary, Dhavan Shah

**Affiliations:** ^1^ Department of Industrial and Systems Engineering Center for Health Enhancement Systems Studies University of Wisconsin-Madison Madison, WI United States; ^2^ Department of Psychiatry School of Medicine and Public Health University of Wisconsin-Madison Madison, WI United States; ^3^ Department of Clinical Sciences College of Health Sciences University of Kentucky Lexington, KY United States; ^4^ Department of Family Medicine and Community Health School of Medicine and Public Health University of Wisconsin-Madison Madison, WI United States; ^5^ Nursing Research Department School of Nursing University of Wisconsin-Madison Madison, WI United States; ^6^ Medical Oncology Section Department of Medicine, School of Medicine and Public Health University of Wisconsin-Madison Madison, WI United States; ^7^ Mass Communication Research Center School of Journalism and Mass Communication University of Wisconsin-Madison Madison, WI United States

**Keywords:** Internet, health communication, palliative care, communication barriers, signs and symptoms, eHealth

## Abstract

**Background:**

Symptom distress in patients toward the end of life can change rapidly. Family caregivers have the potential to help patients manage those symptoms, as well as their own stress, if they are equipped with the proper resources. Electronic health (eHealth) systems may be able to provide those resources. Very sick patients may not be able to use such systems themselves to report their symptoms but family caregivers could.

**Objective:**

The aim of this paper was to assess the effects on cancer patient symptom distress of an eHealth system that alerts clinicians to significant changes in the patient’s symptoms, as reported by a family caregiver.

**Methods:**

A pooled analysis from two randomized clinical trials (NCT00214162 and NCT00365963) compared outcomes at 12 months for two unblinded groups: a control group (Comprehensive Health Enhancement Support System [CHESS]-Only) that gave caregivers access to CHESS, an online support system, and an experimental group (CHESS+CR [Clinician Report]), which also had CHESS but with a CR that automatically alerted clinicians if symptoms exceeded a predetermined threshold of severity. Participants were dyads (n=235) of patients with advanced lung, breast, or prostate cancer and their respective family caregivers from 5 oncology clinics in the United States of America. The proportion of improved patient threshold symptoms was compared between groups using area-under-the-curve analysis and binomial proportion tests. The proportion of threshold symptoms out of all reported symptoms was also examined.

**Results:**

When severe caregiver-reported symptoms were shared with clinicians, the symptoms were more likely to be subsequently reported as improved than when the symptoms were not shared with clinicians (*P*<.001). Fewer symptom reports were completed in the group of caregivers whose reports went to clinicians than in the CHESS-Only group (*P*<.001), perhaps because caregivers, knowing their reports might be sent to a doctor, feared they might be bothering the clinician.

**Conclusions:**

This study suggests that an eHealth system designed for caregivers that alerts clinicians to worrisome changes in patient health status may lead to reduced patient distress.

**Trial Registration:**

Clinicaltrials.gov NCT00214162; https://clinicaltrials.gov/ct2/show/NCT00214162 (Archived by WebCite at http://www.webcitation.org/6nmgdGfuD) and Clinicaltrials.gov NCT00365963; https://clinicaltrials.gov/ct2/show/NCT00365963 (Archived by WebCite at http://www.webcitation.org/6nmh0U8VP)

## Introduction

### Managing Patient Symptom Distress

As advanced cancer treatments enable patients to live longer, managing patient symptoms becomes even more important for patients, informal (family or friend) caregivers, and clinicians [1-5]. In some cases, the side effects of advanced treatment (eg, pain and cognitive limitations) can create problems that challenge the fabric of the family [6,7] and sometimes even lead to conflict [6,7] between the family and the clinical team [8]. Some of the problem, from a patient and caregiver perspective, revolves around when and how to get the attention of the clinical team without “bothering” them [9].

Research has shown the importance of timely alerts to providers regarding disconcerting changes in patient conditions. For instance, a study of wait times in the Veterans Health Administration system found that mortality rates increased significantly when patients waited longer to be seen [10]. However, often changes in a patient’s symptoms are reported only at a clinical visit and by then the patient’s condition is often harder to manage than when the symptoms first worsened.

As early as 1997, family caregivers were estimated to provide about US $200 billion of unpaid health care services [11]. Although the burden on family caregivers has been well described [1], the potential of caregivers to improve their lives, and patients’ and providers’ lives, has had comparatively little attention. When properly engaged, the family caregiver can be a critical source of information and support for both the patient and the clinical team. Family caregivers often spend far more time with the patient than anyone else. They can support the patient while collecting information that could be vitally important to providers and for effective care. Some families assume these roles well with no help, whereas others need support to realize their potential. The system studied here was designed to help caregivers maximize their value as a partner in care and minimize their burden.

Recent developments in electronic symptom collection systems [12,13] offer promise for more timely and accurate information, greater patient acceptance, and reduced cost compared with paper-based systems [14]. Studies of such systems have shown moderate to significant improvement of patient symptoms and quality of life [15-19], and even survival [20]. A key issue is when and how to reach clinicians effectively, given how busy they are.

This problem of reporting symptom changes to clinicians is exacerbated for advanced cancer patients. These patients find it increasingly difficult or impossible to use symptom-reporting systems as their disease advances. Informal caregivers are in a position to accurately observe and report on patient symptoms [21-23].

### The CHESS System With Reports to Clinicians

The Comprehensive Health Enhancement Support System (CHESS) refers to extensively tested information and communication technologies for coping with cancer and other serious illnesses. The vast majority of CHESS systems have been designed for patients [24-27]. The CHESS "Coping with Cancer" website [28] was designed instead for caregivers to (1) provide them with well-organized cancer, caregiving, and bereavement *information*, (2) serve as a channel to *communicate* with and receive *support* from other caregivers, experts, clinicians, and their social networks, (3) act as a *coach* by gathering information from caregivers and providing feedback based on algorithms (decision rules), and (4) provide *tools* (eg, a program to organize support from family and friends) to improve the caregiving experience. Hence the content and focus of this caregiver program differed substantially from other CHESS systems. The relationship between theory and CHESS was previously reported [29].

The CHESS caregiver system studied in this analysis contained a symptom-reporting system, the Clinician Report (CR), which delivered to the clinical team information about worrisome changes in symptoms collected from informal caregivers of advanced-stage cancer patients [29-31]. Specifically, the CR contacted the clinical team whenever a threshold symptom was reported—that is, when a caregiver rated at least one of 10 patient symptoms at ≥7 on a 0 to 10 severity scale. The alert was intended to quickly bring clinician attention to severe symptoms, potentially leading to timelier symptom management.

### Purpose and Contribution of the Study

The study reported here is, to our knowledge, the first to report the effects on patients of an electronic system that collects and analyzes caregiver observations and sends alerts to clinicians when caregivers report worrisome increases in symptom distress in patients suffering from advanced cancer. A previous paper reported the effects on caregivers themselves of using the system [30]. In that study, caregivers with CHESS+CR had less negative mood at both 6 and 12 months than caregivers in the CHESS-Only group. The groups did not differ significantly on caregiver preparedness or physical burden at either time point. This paper reports the effects of the system on patients, specifically on patient symptom distress. We examined two outcomes: (1) the proportion of improved caregiver-reported, severe (“threshold”) symptoms that patients had out of all threshold symptoms and (2) the proportion of caregiver-reported threshold symptoms that patients had out of the total symptoms reported on. Specifically, the study addresses this question: Does the CHESS system with the CR reduce symptom distress in patients more than CHESS without the CR?

## Methods

### Participants

Between September 2004 and April 2007, 235 dyads of patients with advanced-stage cancer and their primary informal caregivers were recruited to one of two randomized clinical trials of CHESS. One of the trials recruited breast and prostate cancer patients and their caregivers (NCT00214162); the other enrolled lung cancer patients and caregivers (NCT00365963). Eligible breast cancer patients were women with recurrent or metastatic breast cancer. Eligible prostate cancer patients had hormone refractory or metastatic prostate cancer. Eligible lung cancer patients included those in stage IIIA, IIIB, or IV disease. Caregivers were at least 18 years of age and identified by the patient as their primary source of physical, emotional, or financial support.

### Recruitment

**Figure 1:**
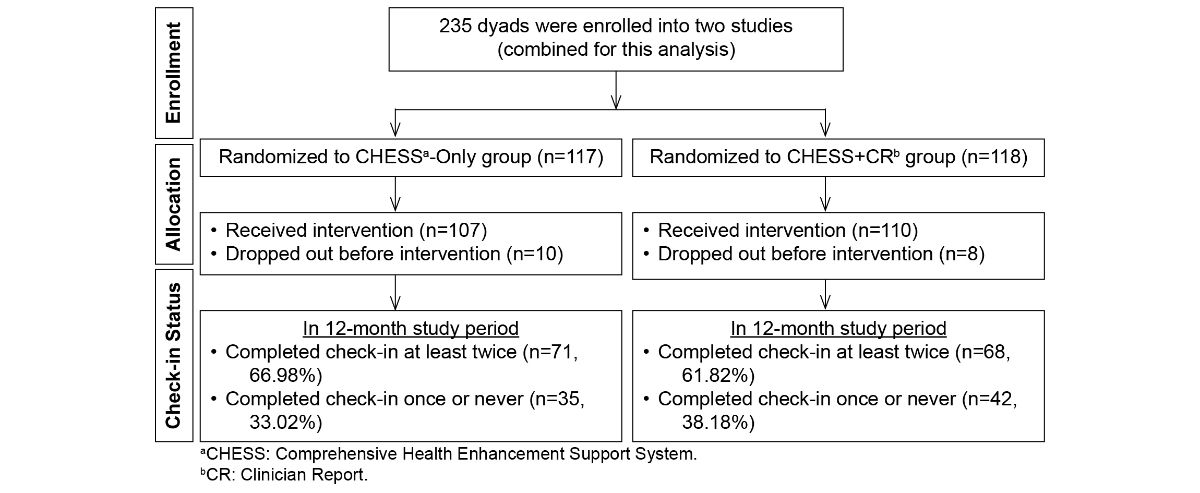
Participant flow.

Recruitment sites were 5 cancer centers in the Northeastern, Midwestern, and Southwestern United States. This analysis contains a combined sample from both clinical trials of 117 dyads in the CHESS-Only group and 118 in the CHESS+CR group (Figure 1). Details about recruitment, randomization, and procedures were previously reported [28,30].

The attrition rate in this study (33.6%, 36/107) in the CHESS-Only group and 38.1% (42/110) in the CHESS+CR group) is comparable with other clinical trials of patients with advanced cancer. A review of 18 interventional supportive and palliative oncology trials found an attrition rate of 44% at study end [32].

### Interventions

Both the CHESS-Only and CHESS+CR participants received access to CHESS. CHESS was designed for caregivers, but patients could have access as well if they wanted it. Upon initial log-in and every 7 days after, caregivers were prompted when logging into CHESS to complete a check-in with questions about patient symptom status from a modified Edmonton Symptom Assessment Scale (ESAS) [33]. Check-in items had to be completed before advancing to another page in the CHESS website. Items on check-ins after the first check-in were populated with previous ratings; caregivers needed to respond only if a rating changed. In the CHESS-Only group, the information reported at check-in was intended for caregivers and not sent to clinicians. In the CHESS+CR group, CHESS summarized the caregiver-provided information and made it available (with patient permission) to the clinical team [31]. Alerts were emailed, faxed, or phoned (according to clinician preference) to a designated member of the clinical team, typically a nurse, (1) when a threshold symptom was reported and (2) 2 days before a scheduled clinic visit. The severity threshold for an alert was set at 7, based on Serlin et al [34], indicating that this symptom distress level interferes significantly with a patient’s life. Alerts included patient name, the symptom or symptoms of concern, and worrisome symptom rating(s), along with a link to the CHESS+CR website to view the complete CR, including ratings over time. At any time, clinicians in the CHESS+CR group could also access the full CR by logging onto CHESS+CR.

### Procedures

The trials were approved by the institutional review boards at each recruitment site. Oncology clinicians were consented and agreed to receive alerts.

After caregivers and patients completed the consent form and pretest, a random number generator at the University of Wisconsin randomly assigned dyads to CHESS-Only or CHESS+CR (1:1 ratio). Randomization was blocked by dyad relationship (spouse or partner vs nonspouse or partner) and race (white vs non-white). All caregivers who needed a computer were mailed a laptop with Internet access and a user manual. Participants who already had a computer with Internet access were reimbursed for Internet access during the study period. Caregivers (and patients, if they wished to use CHESS) were assigned a unique code name and password for accessing CHESS. Technical support was available by telephone. CHESS staff provided training on using CHESS via telephone or in clinic. Those in the CHESS+CR group were told that symptoms reported as “high” would trigger an email to the clinical team. Participants were not told the threshold, but when they gave a rating of ≥7, they were encouraged to call the clinic and notified on the website that the clinical team would be alerted.

Although caregivers and patients were enrolled as dyads, caregivers were the target population in both clinical trials. The analysis uses data from all 12 months of the breast and prostate cancer intervention and, to standardize data from the two trials, from the first 12 months of the 24-month lung cancer intervention. The CHESS website server generated a log file for storing the symptom ratings, who reported the ratings, when the ratings were reported, and whether an alert was sent. These data were retrieved and analyzed for this study.

### Measures

Patient distress is a subjective measure that was assessed at check-in by a modified ESAS [30] on a 0 to 10 scale, with 10 indicating the greatest symptom distress. On the basis of the feedback from oncologists, we replaced 3 physical items in the original scale [33] (activity, drowsiness, and well-being) with three common cancer symptoms (fatigue, constipation, and diarrhea). The modified ESAS contained 10 items. This analysis focuses on individual ratings for each of the 10 symptoms rather than on a single scale score calculated across symptoms.

Improvement was determined separately for each threshold symptom by comparing subsequent ratings given by the same person at check-in. A threshold symptom was considered improved if it was rated lower at the next check-in (eg, if a caregiver rated patient pain at 9 one week and 8 the next week, the symptom was considered improved; likewise, a symptom rated at 7 one week and 6 the next was considered improved). Patient symptom ratings given in the last check-in were not examined because no follow-up check-in could be compared with it. Patients and caregivers who completed fewer than two check-ins were excluded in the analysis because they supplied no data for comparison.

To evaluate the impact of the CR on symptom change, caregiver-reported check-in data were aggregated in six 2-month periods (eg, months 3 and 4). This interval is somewhat arbitrary. It was chosen because caregivers in the two randomized trials from which the data came filled out written surveys every 2 months. The following values were calculated: (1) number of assessed symptoms (number of discrete symptoms rated during a 2-month period, calculated as the number of times a check-in was completed multiplied by 10 because each check-in assessed 10 symptoms), (2) number of patient threshold symptoms (number of symptoms rated ≥7), and (3) number of improved patient threshold symptoms (number of threshold symptoms with a lower rating in the next check-in). Group totals of the foregoing three variables were used to calculate the following two proportion indices for each 2-month period as well as the entire 12-month study period. Our primary interest was the proportion of improved caregiver-reported patient threshold symptoms (number of improved threshold symptoms out of the total number of threshold symptoms, which shows the impact on symptom management). We also calculated the proportion of caregiver-reported patient threshold symptoms (number of caregiver-reported threshold symptoms out of the total number of symptoms that were reported on). The purpose of the second proportion was to examine whether knowledge that their data might be reported to the clinical team would affect reporting behavior.

### Statistical Analysis

To compare the two outcomes of interest between groups over time (ie, improved patient threshold symptoms and proportion of patient threshold symptoms), the area under the curve (AUC) was calculated based on group-aggregated values. The AUC per group was calculated using the trapezoidal rule in NCSS 2007 [35]. Group differences in AUC were assessed by converting group AUC into a relative proportion per group and then conducting a proportional difference test (StatXact 5, Cytel) [36]. In addition, aggregated symptom reports were averaged across the 12 months. Differences in these averaged improved threshold symptoms and proportions of threshold symptoms were tested using the same method. The standardized statistics, *P* value and 95% CIs, were calculated based on methods outlined by Miettinen and Nurminen [37] and Chan and Zhang [38]. To test for potential response bias after group assignment, the proportion of patient threshold symptoms reported by caregivers at pretest was compared with the proportion at the first check-in using the multiple-sample McNemar test [39]. All tests were conducted at alpha=.05 level.

## Results

### Baseline Demographics and ESAS Ratings

Table 1 shows demographics and pretest ESAS ratings for the two groups. Among patients, 55.8% (121/217) were female, with an average age of 63 years. Caregivers were predominantly female (64.2%, 140/217), with an average age of 56 years. Most caregivers (69.3%, 150/217) were spouses or partners. Demographic characteristics of caregivers omitted from the analysis (ie, caregivers who submitted fewer than two check-ins) were similar to those of caregivers included in the analysis except on caregiver gender, for which 53% of excluded caregivers and 71% of included caregivers were female (χ^2^_1_=6.4 *P*=.01). In the 12-month period, the proportion of patient symptoms reported by caregivers at least twice did not differ by randomization group (CHESS-Only, 71/107, 66.4% vs CHESS+CR, 68/110, 61.8%, standardized difference Z=0.696, *P*=.49, 95% CI −.082 to .171).

**Table 1:** Demographics of participants who received the interventions.

Characteristics			CHESS^a^-Only (n=107)		CHESS+CR^b^ (n=110)	
**Cancer type, n (%)**						
	Breast		45 (42)		44 (40)	
	Prostate		30 (28)		34 (31)	
	Lung		32 (30)		32 (29)	
**Patients**						
	Age, mean (SD)		62.53 (9.63)^c^		62.73 (11.00)^d^	
	**Gender, n (%)**					
		Male		47 (44)		49 (45)
		Female		60 (56)		61 (55)
**Caregivers**						
	Age, mean (SD)		55.73 (13.02)^c^		56.36 (13.39)^e^	
	**Gender, n (%)**					
		Female		71 (66)		69 (63)
	**Relationship to patient, n (%)**					
		Spouse		75 (70)		75 (68)
		Nonspouse		32 (30)		35 (32)
	**Caregiver annual household income in US dollars, n (%)**					
		Below $40,000		35 (33)		35 (32)^c^
		$40,001-$80,000		37 (35)		36 (33)
		$80,001 and over		26 (24)		28 (25)
		Didn’t report		9 (8)		11 (10)
	Caregiver education (1-6)^f^, mean (SD)		3.96 (1.58)^g^		3.67 (1.52)^d^	
	Caregiver Internet comfort (1-4)^h^, mean (SD)		2.57 (1.26)^i^		2.36 (1.37)^e^	
	Caregiver-reported patient ESAS^j^ (1-90)^k^, mean (SD)		27.75 (16.82)^l^		28.13 (15.90)^i^	

^a^CHESS: Comprehensive Health Enhancement Support System.

^b^CR: Clinician Report.

^c^n=105.

^d^These values were based on pretests of 109 caregivers in CHESS+CR group because 1 caregiver did not return the pretest survey.

^e^n=107.

^f^Caregiver education levels: 1 Stopped school before finishing high school; 2 High school degree; 3 Some college courses; 4 Associate or technical degree (2-year college); 5 Bachelor’s degree (4 year college); 6 Graduate degree.

^g^n=106.

^h^Internet comfort levels: 0 Not at all; 1 A little; 2 A medium amount; 3 Quite a bit; 4 Extremely.

^i^n=102.

^j^ESAS: Edmonton Symptom Assessment Scale.

^k^Calculated as the sum of severity ratings (0=none; 10=worst possible) across 9 items.

^l^n=94.

^m^n=101.

**Table 2:** Bimonthly summary of caregiver-reported patient symptom indices. At pretest, ESAS items were rated via paper survey. The report of a threshold symptom did not produce a clinician alert.

Randomization group	CHESS^a^-Only	CHESS+CR^b^
Pretest	117	118
Month 2	101	104
Month 4	96	93
Month 6	88	85
Month 8	78	74
Month 10	72	68
Month 12	65	63

^a^CHESS: Comprehensive Health Enhancement Support System.

^b^CR: Clinician Report.

We also examined data about participant use of the system to see whether different participants used the system differently (ie, by gender, race, age, living situation, education, employment status, or income) and found no statistically significant difference.

### Symptom Indices and Proportion of Threshold to All Assessed Symptoms

Table 2 shows patient symptom indices reported by caregivers calculated for each 2-month period and totaled across 12 months. Table 3 reports on the number of patient threshold symptoms reported by caregivers as a proportion of all assessed symptoms. At pretest, for instance, CHESS-Only caregivers reported on symptoms 1026 times, and 182 of those symptoms (17.74%) met or exceeded the threshold. CHESS+CR caregivers reported 1056 symptoms, and 184 (17.42%) were threshold symptoms. The effect size of the difference between CHESS-Only and CHESS+CR was 0.01. Effect sizes were determined by using Cohen arcsine transformation of the probabilities [40]. At pretest, there were no significant differences between groups, but differences emerged (caregivers in CHESS+CR reported fewer symptoms) with what Cohen describes as a small effect size.

### Proportion of Improved Threshold Symptoms to All Threshold Symptoms

Table 4 reports on the number of improved threshold symptoms as a proportion of all threshold symptoms reported. For instance, at 2 months, the CHESS-Only caregivers reported 385 threshold symptoms, 103 of which (26.8%) were subsequently reported as improved. By contrast, at the same time point, CHESS+CR caregivers reported 212 threshold symptoms, 113 of which (53.3%) were subsequently reported as improved. Comparing CHESS vs CHESS+CR across all posttest time periods yielded a moderate effect size of 0.60 in favor of CHESS+CR.

Analyses of average aggregated proportions across all 12 months (the Total column of Tables 3 and 4) show similar findings: the CHESS+CR group was more likely to report improvement (53.04% vs 26.16%, Wald Z-test=10.35, *P*<.001, 95% CI .216 to .320) but less likely to report threshold symptoms (7.7% vs 14.4%, Wald Z-test=−12.27, *P*<.001, 95% CI −077 to −057). That is, throughout the study period, caregivers in the CHESS+CR group consistently reported that their patients had less symptom burden and better symptom management than patients in CHESS-Only group.

**Table 3:** Proportion of threshold symptoms/all assessed symptoms and effect sizes.

Randomization group	CHESS^a^-Only, n/N (%)	CHESS+CR^b^, n/N (%)	Effect size (95% CI)
Pretest	182/1026 (17.74)	184/1056 (17.42)	0.01 (0.00-0.02)
Month 2	385/2620 (14.69)	212/2380 (8.91)	0.18 (0.17-0.19)
Month 4	263/1820 (14.45)	83/1240 (6.70)	0.26 (0.23-0.28)
Month 6	219/1490 (14.70)	74/840 (8.81)	0.18 (0.16-0.21)
Month 8	133/1140 (11.67)	34/550 (6.2)	0.19 (0.14-0.25)
Month 10	140/910 (15.38)	26/390 (6.7)	0.28 (0.21-0.36)
Month 12	129/830 (15.54)	14/350 (4.0)	0.41 (0.27-0.55)
Total	1269/8810 (14.40)	443/5750 (7.70)	0.216 (0.221-0.220)

^a^CHESS: Comprehensive Health Enhancement Support System.

^b^CR: Clinician Report.

**Table 4:** Proportion of improved threshold symptoms/all threshold symptoms and effect sizes.

Randomization group	CHESS^a^-Only, n/N (%)	CHESS+CR^c^, n/N (%)	Effect size (95% CI)
Pretest	N/A^b^	N/A	N/A
Month 2	103/385 (26.8)	113/212 (53.3)	0.55 (0.52-0.57)
Month 4	67/263 (25.5)	44/83 (53)	0.57 (0.52-0.62)
Month 6	53/219 (24.2)	41/74 (55)	0.60 (0.55-0.66)
Month 8	39/133 (29.3)	17/34 (50)	0.42 (0.30-0.56)
Month 10	33/140 (23.6)	14/26 (54)	0.64 (0.48-0.79)
Month 12	37/129 (28.7)	6/14 (43)	0.30 (0.04-0.64)
Total	332/1269 (26.16)	235/443 (53.0)	0.60 (0.59-0.62)

^a^CHESS: Comprehensive Health Enhancement Support System.

^b^N/A: not applicable.

^c^CR: Clinician Report.

### Examination of Possible Response Bias

Caregivers in the CHESS+CR group reported a lower proportion of threshold symptoms at each 2-month period (Table 3). It may be that they felt they might be bothering the doctor or that word would get back to the patient that the caregiver thought the patient was doing poorly. To examine whether caregivers ranked symptoms lower to avoid triggering the alert to the clinician, we compared symptom ratings at pretest with those at the first online check-in, assuming any reluctance to alert the clinician would not have been present on pretest because pretest data were not sent to clinicians and patients knew this. A multiple-sample McNemar test [39] was used to test for differences between the CHESS-Only and CHESS+CR caregiver groups on changes in reporting threshold symptoms from pretest to first online check-in. A total of 1329 symptoms were rated by 135 caregivers (70 CHESS-Only, 65 CHESS+CR) at pretest and first online check-in and were used in this analysis. At pretest, no significant difference was found between randomized groups in the number of patient threshold symptoms reported (Z=0.189, *P*=.85, 95% CI −0.03 to 0.04), but the difference in reporting threshold symptoms from pretest to first online check-in was statistically significant (Z=6.910, *P*<.001, 95% CI 6.50 to 7.29), with CHESS+CR caregivers reporting a lower proportion of threshold symptoms at first check-in than at pretest (Table 5), suggesting that CHESS+CR caregivers may have had a response bias toward lower ratings when they knew a clinician might be alerted.

Further analyses also suggest that bias may help explain the results. First, we examined the relationship between caregiver demographics and the likelihood of reporting symptom data in the CHESS-Only versus CHESS+CR groups. Gender was the only statistically significant characteristic related to the reporting of symptoms: 64.7% (172/266) of women are reporters overall versus 49.4% (131/266) of men; χ^2^_1_^ ^(N=266)=5.2, *P*=.02. In the CHESS+CR group, 64.4% (75/117) of women versus 45.5% (53/117) of men were reporters, χ^2^_1_^ ^(N=117)=4.02, *P*=.045; no significant differences were found in the CHESS-Only group. We also looked at 35 outcomes, such as caregiving burden and patient quality of life at each survey time frame, and did not find a consistent difference between caregivers who reported symptoms and those who did not.

We also examined, in addition to improved symptoms, which were the focus of the study, the proportion of symptoms that stayed at the same level of severity and the proportion that worsened. Caregivers in the CHESS-Only group reported more total symptoms (8810) and a greater proportion of threshold symptoms (14.40%, 1269/8810) than caregivers in the CHESS+CR group (5750 total symptoms, of which 7.70% [443/5750] were threshold symptoms). Caregivers in the CHESS+CR group reported a much higher percentage of improved threshold symptoms (53.0% [235/443] versus 26.16% [332/1269] in the CHESS-Only group), a slightly larger percentage of worsened symptoms (14.0% [62/443] versus 10.40% [132/1269] in the CHESS-Only group), and a much lower percentage of threshold symptoms with no change (33.0% [146/443] versus 63.4% [805/1269] in the CHESS-Only group).

Finally, we looked at how many caregivers reported symptoms in the 2 groups. Over the 12 months, 71 caregivers reported the 8810 symptoms in the CHESS-Only group compared with 68 caregivers in the CHESS+CR group, who reported on 5750 symptoms. On average, each reporting caregiver in the CHESS-Only group reported on 124.1 symptoms whereas caregivers in CHESS+CR group reported an average of 84.6 symptoms. Hence, a 31.8% difference exists between the groups in the average number of symptoms reported per caregiver, with those in the CHESS+CR group reporting fewer.

**Table 5:** Proportion of patient threshold symptoms reported by caregivers at pretest versus initial check-in.

Randomization group	CHESS^a^-Only, n/N (%)	CHESS+CR^b^, n/N (%)	Effect size (95% CI)	*P* value
Pretest	106/684 (15.5)	105/645 (16.3)	0.02 (0.00-0.04)	.85
Initial check-in	108/684 (15.8)	70/645 (10.9)	0.15 (0.12-0.18)	<.001

^a^CHESS: Comprehensive Health Enhancement Support System.

^b^CR: Clinician Report.

## Discussion

### Principal Findings

The CR was designed to speed information about patient symptoms to clinicians by automatically sending an alert when a caregiver reported a symptom to be at or over a threshold. With immediate symptom reporting, clinicians can intervene rapidly and reduce patient symptom distress. Our results show that for symptoms causing severe (≥7 on a 0 to 10 scale) distress, patients whose caregivers had access to CHESS+CR, and therefore had an alert sent to their clinicians, had a greater proportion of symptom improvements than those with CHESS-Only, whose clinicians did not receive alerts or have access to ratings. These results suggest that the CR may facilitate patient symptom improvement and management. A previously published paper [30] found that the same system, CHESS+CR, improved caregivers’ negative mood, suggesting that one electronic health (eHealth) system may help both caregivers and indirectly the patients themselves.

### Possible Explanations of the Results

The effects of the CR may be explained in various ways, as clinicians explained in qualitative interviews [31], which include the following:

1. The CR could help clinicians better prepare to address patient symptoms and caregiver concerns in clinic visits.
2. The CR may boost caregiver efficacy in discussing symptoms with clinicians.
3. The CR may deepen caregiver involvement because caregivers can monitor patient symptoms and report their concerns directly to the clinical team.
4. The CR may enable earlier intervention because the severe symptom distress that triggers alerts might otherwise be unreported and therefore not attended to.

The overall assessed symptom rate in the CHESS+CR group dropped significantly more than in the CHESS-Only group. A response bias may have occurred if caregivers avoided using the check-in or rated symptoms lower because they feared bothering the clinician [41] or upsetting the patient. The finding that symptom distress was equal between the two study groups at pretest but lower in the CHESS+CR group at first check-in supports this explanation. The examination of other factors that may account for the difference between the groups in caregiver-reported symptoms (ie, demographic differences between caregivers in the two groups, the proportion of symptoms that stayed the same and worsened in each group, and the number of caregivers reporting symptoms in each group) corroborates the suggestion of bias among the CHESS+CR caregivers to report fewer symptoms.

The lower overall assessed symptom rate in the CHESS+CR group may also be explained by clinician response. Clinicians were given no directions about how to respond to CR alerts. If clinicians did not respond (we do not know whether they did), participants may have stopped using the CR, although the data suggest that when the CR alerts the clinician, the symptoms are more likely to improve. It may be that clinicians responded to alerts by addressing the symptoms more promptly, scheduling additional patient visits, or responding differently to patients during visits, though we lack the data to explore this. Future research on caregiver and patient motives for using or not using a symptom reporting system could better inform system development and dissemination.

### Limitations

The study has limitations. Data collection was completed in 2009. We do not see this as a major limitation because the influence of caregiver input on clinical decision making is rarely examined, and the result of such input, as shown in this paper, can be significant for patients. In fact, because reports based on the regular collection of both patient and caregiver data are beginning to appear more frequently, the methods and results reported here are more likely to be timely now than several years ago.

Each institution had a small number of clinicians, and they were not randomized. The effect of the CR on symptom improvement could potentially be greater than demonstrated here because clinicians seeing patients in both groups may have improved their symptom management with control-group patients as a result of changes they made with the intervention group. Future research could avoid this by randomizing clinics or clinicians rather than caregiver/patient dyads within clinics, although this approach could confound clinic variability in care with randomization.

Furthermore, all outcome variables were self-reported. Medical records could have been used to validate self-reports, for example, by observing whether interventions seemed to respond to reported threshold symptoms, such as a change in the type or amount of medication if high pain levels were reported. Such an analysis was outside the scope of the study.

Although clinicians participated in qualitative interviews [31] (results are summarized above under Possible Explanation of the Results), a distinct weakness of the paper is the lack of qualitative evidence from caregivers and patients. For example, qualitative assessment could also have been used to explore the apparent bias in the CHESS+CR group about “bothering” the doctor.

Although the study suggests the potential of CR-like systems to enhance patient care and speed recovery from distressing symptoms, further research with different patient populations would help validate and improve the generalizability of these findings. In addition, widespread use of such a system poses important challenges—cost, risk aversion, clinician time, and interoperability with the electronic health record (EHR). Caregivers could use such a system on their smartphones, which are becoming ubiquitous. Yet maintaining such an eHealth system has fixed costs (eg, for updating content and moderating discussions). These costs could be borne by health systems and insurers if outcomes warrant it, given the new financing models that reward governmental agencies to pay for systems that improve outcomes and the increasing use of fixed payments to providers. The human tendency to avoid risk and stay with the familiar works against innovations such as CR-like systems, as does the time pressure clinicians contend with. Because clinicians worry about innovations that add work and reduce time with patients, systems must be built to minimize burden. Finally, as we have discovered in subsequent studies, getting information from such a system into the EHR can be extremely difficult. However, this is important and should be done so that clinicians can access information from patient and caregiver eHealth systems without going to a website outside the EHR.

### Conclusions

The significance of this study is its finding that eHealth support helped caregivers play a more effective role in their loved one’s care than the role they played without that support. Furthermore, the results suggest that eHealth alerts coming from family caregivers can influence clinician behavior. Together, these findings suggest that eHealth-based CRs from caregivers can influence care for patients with many types of cancer, as well as chronic conditions such as metabolic syndrome, addiction, human immunodeficiency virus, and Alzheimer disease [42,43]. Further research is required to address this speculation.

## Abbreviations

AUC: area under the curve
CHESS: Comprehensive Health Enhancement Support System
CR: Clinician Report
eHealth: electronic health
ESAS: Edmonton Symptom Assessment System
EHR: electronic health record
NIH: National Institutes of Health

## Appendix

Multimedia Appendix 1 CONSORT‐EHEALTH checklist (V.1.6.1).
